# Medical nutrition therapy in Canadian federal correctional facilities

**DOI:** 10.1186/s12913-019-3926-3

**Published:** 2019-02-01

**Authors:** Karen M. Davison, Carla D’Andreamatteo, Victoria L. Smye

**Affiliations:** 1 School of Nursing, University of British Columbia, Vancouver, BC Canada; 2 Fulbright Canada Visiting Research Chair, College of Social Sciences, University of Hawai’i at Mānoa, Honolulu, Hawaii USA; 3 Department of Biology, Health Science Program, Kwantlen Polytechnic University, Surrey, BC Canada; 4 Food and Nutritional Sciences, University of Manitoba, Winnipeg, Manitoba Canada; 5 Arthur Labatt Family School of Nursing, Western University, London, Ontario Canada; 6 Health Science, University of Ontario Institute of Technology (UOIT), Oshawa, Ontario Canada

**Keywords:** Medical nutrition therapy, Nutrition, Dietitian, Incarceration, Prison, Qualitative study

## Abstract

**Background:**

Under- and over nutrition as well as nutrition risk factors such as communicable and non-communicable diseases are a common and major cause of morbidity and mortality in correctional facilities. Consequently, medical nutrition therapy (MNT), a spectrum of nutrition services aimed at optimizing individual well-being, is being recognized as integral to the health of people who experience incarceration. However, there is a paucity of research that explores the delivery of MNT in correctional facilities.

**Methods:**

A scoping review combined with secondary analysis of qualitative data (field notes, in-depth stakeholder interviews) from a 2-year ethnographic study about food insecurity and incarceration was undertaken to gain insights about the delivery of corrections-based MNT in Canada. Thematic analysis of all documents was done using an interpretive framework.

**Results:**

An understanding about MNT was developed within three themes: 1) specialized service provision in a unique environment; 2) challenges with the provision of MNT; and 3) consideration of corrections-based MNT alternatives. An incarcerated individual’s nutritional health was conceptualized as culminating from various factors that included dietary intake and health status, enabling environments, access to quality health services, and clinical nutrition services. Nutrition care practices, which range from health promotion to rehabilitation, are challenged by issues of access, visibility, adequacy, and environmental barriers. Their success is dependent on demand (e.g., ability of recipient to act) and factors that enable quality health and food services. Advancing corrections-based MNT will require policies that provide supportive food and health environments and creating sustainable services by integrating alternatives such as peer approaches and telehealth.

**Conclusions:**

Professional associations, government, researchers and other stakeholders can help to strengthen corrections-based MNT by fostering shifts in thinking about the role of health practitioners in these contexts, preparing future health professionals with the specialized skills needed to work in these environments, generating evidence that can best inform practice, and cultivating collaborations aimed at crime prevention, successful societal reintegration, and the reduction of recidivism.

**Electronic supplementary material:**

The online version of this article (10.1186/s12913-019-3926-3) contains supplementary material, which is available to authorized users.

## Background

Compared to general populations, people who experience incarceration have poorer physical, mental, and social health, making them among one of the most marginalized populations [1–5]. Many incarcerated persons arrive at correctional facilities with histories of inadequate access to preventive care and health services for acute or chronic health problems. Malnutrition, that encompasses both under nutrition and over nutrition [6], is common and a major cause of morbidity and mortality both in correctional facilities of developed and less developed countries [3, 7].

It is well established that nutrition is a determinant of physical and mental health and well-being [8]. Indicators of poor nutrition, such as high sodium intakes and dyslipidemia, and dietary risk factors such as substance use, infectious and chronic conditions are prevalent among incarcerated persons [3–7, 9–14]. Providing appropriate nutrition and food services as a form of duty of care has the potential to generate critical benefits including the prevention of communicable diseases that poses threats to community health, reducing risk of developing non-communicable health conditions and associated cost burdens, improving chronic condition management, reducing problematic and self-harm behaviors, and preventing recidivism [3, 9, 11, 14–16].

Food and nutrition services within correctional facilities of developed countries generally have two interdependent areas of delivery [17, 18]. The first, often referred as the administrative component, involves the development and implementation of standards, menu planning, food service staff training, budget control, and food policy standards, as well as systems that are foundational to institution activities. The second component involves medical nutrition therapy (MNT), a spectrum of nutrition-related diagnostic, therapy, and counseling services aimed at health and disease management [19]. MNT is often delivered by a registered dietitian or nutrition professional who works as part of an interdisciplinary health team. Examples of tasks executed by a MNT practitioner would include nutrition assessment and care planning, collaborative goal setting, implementation and evaluation of an individual’s nutrition care plan, interprofessional nutrition education, and advocacy. While nutrition services are generally classified into these two components which are often managed separately, they are integrated in various ways such as in the planning of departmental resources (e.g., food, equipment, type of service delivery), service provision, and policies. Many investigations that focus on nutrition service delivery and incarceration are largely based on food services systems, particularly factors such as food adequacy and quality [20, 21]. However, investigations of corrections-based MNT have been limited, despite reports that there are significant challenges in practice (e.g., limited resources, working with individuals that have several co-morbidities) [3, 11, 15]. A deepened understanding of the relationships and contextual factors that impact MNT in correctional systems could provide information to improve food and nutrition programs, practices, and policies which, in turn, could reduce health care and criminal justice system costs.

Several information gaps about the implementation, associated challenges, and effectiveness of corrections-based MNT exist. While there have been studies of the administration of food and nutrition services, few discuss the interface with clinical services or the experiences in other developed nations besides the United States, United Kingdom, and Australia. In particular, there is a paucity of research from Canada, which has unique correctional service features related to the dispersion and organization of nutrition services. Canadian-based data contributions that include examination of the interconnections between administrative and clinical food and nutrition services could serve to inform global discussions about nutritional status and incarceration. Furthermore, studies aimed at providing information about how healthy environments in Canadian correctional facilities can be created have been identified as a research priority for prison health [22].

In Canada, an ethnographic study that focused on food insecurity and incarceration was undertaken. This investigation included naturalistic observation-based field notes about food provision and nutrition services in correctional facilities. In addition, the study included in-depth individual and focus interview data from individuals who were currently or formerly incarcerated and stakeholders involved in corrections and societal reintegration services. The availability of this detailed data provides a rare opportunity to examine the delivery of MNT in Canadian correctional facilities from the perspectives of multiple stakeholders.

## Methods

### Study context

This study was a secondary analysis of data from a qualitative study that focused on food and nutrition systems in federal level correctional facilities (i.e., houses offenders serving a sentence of ≥ 2 years) and barriers/facilitators of food security experienced by incarcerated individuals [23]. The study location was in the Fraser Valley region of British Columbia and the data was derived from 24 months of ethnographic fieldwork, including in-depth interviews from various stakeholders.

### Study rationale

In Canada, there are more than 14,500 adults in custody in federal institutions and more than 8000 supervised by Correctional Service Canada (CSC) in the community [24]. Relative to the Canadian population distribution, a higher proportion of individuals less than 35 years of age (> 50%) and that are Aboriginal (25%) are admitted to sentenced custody [25, 26]. The health and social characteristics of those in custody have been described as having histories of childhood traumas, low socioeconomic status, *Diagnostic and Statistical Manual of Mental Disorders* diagnoses (i.e., substance use disorders, antisocial personality, affective disorders, schizophrenia, cognitive impairment, eating disorders), communicable diseases (e.g., tuberculosis, hepatitis C, HIV, sexually transmitted infections) and chronic conditions (e.g., cardiovascular disease, diabetes, asthma and other respiratory diseases, epilepsy) [14, 27–30]. While recent data about health service use prior to incarceration is lacking, prescription medication use during incarceration is high, particularly for psychotropic medications [14]. Furthermore, mortality rates, generally due to homicide or suicide, for persons in custody exceed that of the general Canadian population [14, 31]. Thus, it is evident, that Canadians in correctional facilities face multiple health and social challenges, however, appropriate health interventions while in custody could provide the opportunity to initiate health behavior changes that would lead to important societal benefits.

### Study phases

The investigation included two phases:

#### Phase one: Document analysis and naturalistic observation

Documents about incarceration and reintegration (e.g., policies, legislation) were reviewed. Two researchers (one dietitian/researcher and one social worker/researcher) conducted naturalistic observation [32] over 16 months at the sites of two community agencies that provide services to current and formerly incarcerated individuals and during site visits to federal correctional facilities.

#### Phase two: In-depth individual and focus group interviews

Purposive and theoretical sampling was used to gather in-depth interview data about food provision practices and incarceration from a diversity of participants (*n* = 63; Table 1) including nutrition practitioners (registered dietitians) who were working in a federal correctional facility or had worked in these settings (*n* = 6). All study participants provided written informed consent prior to individual and focus group interviews. With permission, interviews were audio-taped and transcribed. All aspects of the research received ethics approval by the Behavioral Ethics Review Board at the University of British Columbia (reference number H12–02095). The interview guides used for stakeholders and those with lived experience of incarceration are located in Additional file 1.

**Table 1:** Description of Participants (*n* = 63)

Description	# of people
In-Depth Individual Interviews	
Stakeholders^a^: ᅟ• Corrections system (*n* = 16); included 6 dietitians who work (current or previous) in corrections ᅟ• Reintegration (*n* = 13)	29
Individuals with lived experience of incarceration	11
Partners of individuals with lived experience of incarceration	7
In-Depth Focus Group Interviews	
Three focus groups that included: ᅟ• Individuals with lived experience of incarceration (*n* = 2) ᅟ• Partners of individuals with lived experience of incarceration (*n* = 3) ᅟ• Stakeholders^a^ (*n* = 11) within the corrections system (*n* = 3) and involved in societal reintegration (*n* = 8)	16

^a^Examples: Long-term Inmates Now in Community (L.I.N.C), Elizabeth Fry Society, The John Howard Society of Canada, St. Leonard’s Society, Correctional Service Canada, Hope Central, registered dietitians who currently or formerly worked in corrections

### Use of secondary data

Secondary data analysis provides an efficient way to re-examine previously collected information to generate new insights and knowledge [33]. Ethical questions have been raised about the distance between secondary researchers and primary data when the researchers have not been involved in data collection [33]. However, in this study the same researchers analyzed the data from both the first and second investigation. Furthermore, the data collected from naturalistic observations provided context to better comprehend stakeholder perspectives [33].

### Analysis

The secondary analysis for this study was conducted in three stages:

#### Scoping review

A scoping review [34] was conducted by two research team members (KD, CD’A) to examine existing literature (1995 to present) about corrections-based MNT in developed countries. The review included keyword searches (i.e., diet*, nutr*, food, incarc*, jail, pris*) of various databases: Medline, PsycINFO, Embase, Social Sciences Abstracts, Sociological Abstracts, CINAHL, Criminal Justice Abstracts, ERIC, Proquest Criminal Justice, Proquest Dissertations and Theses, Web of Science, and Scopus. In addition, non-indexed sources were searched that included the Journal of Foodservice and The Journal of Foodservice Management and Education and reference lists of included studies and relevant reviews were scanned. The websites of relevant organizations such as Correctional Service of Canada, the Office of the Correctional Investigator, The John Howard Society of Canada, and the Canadian Association of Elizabeth Fry Societies were also reviewed. Any documents that directly or indirectly discussed MNT in correctional facilities (e.g., programs, services, recommendations) were included.

#### Thematic analysis

Interpretive approaches [35, 36] grounded in a systems view were applied. The underlying assumptions were that nutrition services are an ‘intervention’ within systems and in-depth exploration of these services will create opportunities to enable capacity building [37]. MNT was conceptualized as a process of interlinked components that included individual experiences, service implementation, health and social services contexts, the corrections environment, and societal reintegration.

Using all of the original uncoded field notes and transcripts, manual thematic analysis was done by two research team members (KD and CD’A) to produce insights into MNT processes and their inter-relationships within the correctional system’s environment. To help ensure rigor, stakeholder and scoping review data were compared for consistency and thematic results were reviewed by dietitians and staff (e.g., food service employees, health professionals) with correctional experience and formerly incarcerated individuals to verify that the accounts were representative and comprehensive [38].

## Results

### Scoping review

#### Description of corrections-based health and nutrition Services in Canada

The standards for health care in Canadian federal correctional facilities are outlined in the *Corrections and Conditional Release Act* [39]. In Canadian federal corrections, the policy direction for the delivery of food services is the responsibility of the Assistant Commissioner, Corporate Services. The Assistant Warden, Management Services provides general direction for the delivery of food services. The Food Services Chief directly coordinates and manages all food services activities [40] with the assistance of registered dietitians.

**Fig. 1:**
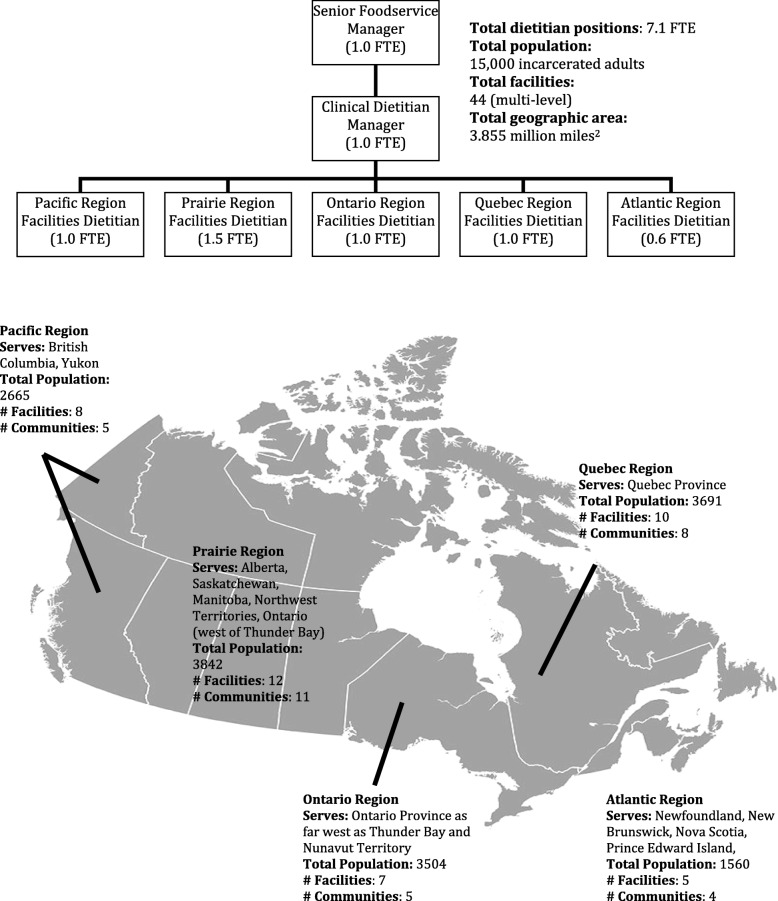
Overview of nutrition services in Canadian federal correctional facilities [110]

At the time of the study, CSC employed eight registered dietitians with a workload of 7.1 full time equivalents (Fig. 1). Approximately 15,000 incarcerated adults (~ 2150 incarcerated individuals per one FTE dietitian) rely on CSC for their nutrition care and daily food. The dietitians, assigned by region, are part of interdisciplinary teams that include physicians, nurses (~ 700), psychologists and assistant psychologists (~ 300), pharmacists, and social workers [41]. While the ratio of dietitians to incarcerated individuals may appear to be favorable, unlike other countries such as the US (~ 13,800 incarcerated individuals per one FTE dietitian) [42], Canadian federal corrections facilities do not employ nutrition and dietetic technicians that work collaboratively with dietitians [43].

#### Results of scoping review

**Fig. 2:**
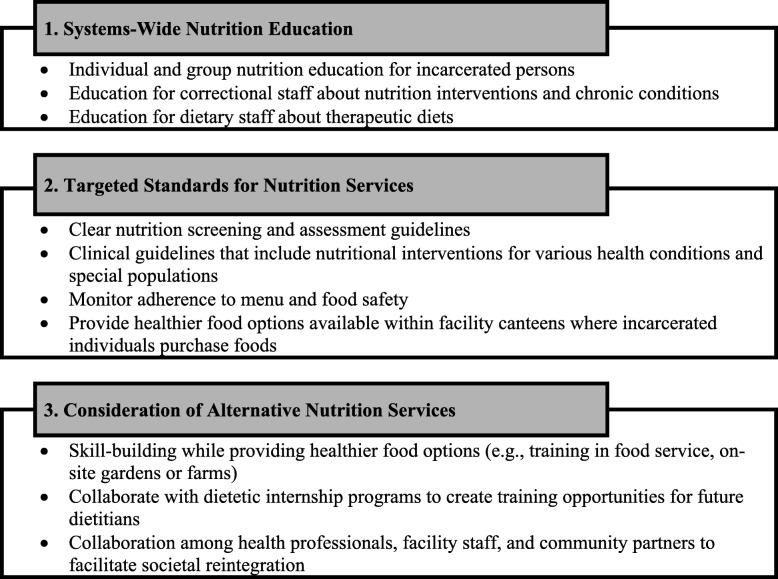
Themes derived from scoping review

The searches yielded 241 resources. Of these, 49 were included that either directly or indirectly discussed corrections-based MNT (1996 to 2017): two were from Canada [44, 45], 30 from the United States [46–75], and 16 from other developed nations, primarily the United Kingdom and Australia [7, 9, 11, 20, 21, 76–87]. Thematic analysis of the screened resources highlighted three broad areas where corrections-based MNT could be more effective (Fig. 2): 1) systems-wide nutrition education; 2) targeted standards for nutrition services; and 3) consideration of alternative nutrition services models. Further details and examples from these screen documents are integrated in the thematic analysis results.

### Thematic analysis of naturalistic observation and interview textual data

Most of the individual and focus group interview data provided direct or indirect information and perspectives related to corrections-based food and nutrition services. Stakeholder interview data primarily included perspectives about these services from health and food services staff (*n* = 6), incarcerated individuals (*n* = 7) and their family members (*n* = 3), and persons whose work focused on societal reintegration (*n* = 9) (e.g., employees of transitional houses and community agencies, parole officers). From the analysis three themes emerged: 1) specialized service provision in a unique environment; 2) challenges with MNT provision; and 3) consideration of corrections-based MNT alternatives.

Theme 1: Specialized Service Provision in a Unique Environment.

Consistent with the research literature that describes the importance of food in correctional facilities in relation to physical, mental, and social health [84], stakeholders discussed how food is central to the life of an incarcerated individual [7]:

“*As far as food..for many it’s all they got.” (Stakeholder Incarceration; SI#1).*

*“..food was a big deal. So to change things like taking pizza off the menu … could lead to riots. So you had to be really careful with some of the choices you made around the menu.” (Dietitian#6; RD#6)*

Most tended to discuss the quality of food and overall food environment. Individual nutrition counseling was the main area where direct MNT services were provided. The most frequent topics were type 2 diabetes and weight management which is common in this population [74] and are part of core competencies of entry-level dietitians. However, there are a variety of health issues such as hepatitis C, HIV/AIDS, tuberculosis, cardiovascular disease, mental health conditions, long-term neurological conditions, renal disease, substance use, complex behaviors, and historical trauma [79, 80] that are common. In addition, overrepresentation of population characteristics such as poverty, unemployment, and Aboriginal ethnicity may require higher level practitioner competencies [88, 89].

*“It is a challenging job … it’s a really difficult population to work with..I remember running into one inmate who had been in segregation for a really long time..he couldn’t remember how to follow recipes, he couldn’t remember how to cook foods anymore..and I had just started working..and I was like don’t worry all you have to do is Google simple recipes … he looked at me like I had no idea what I was talking about..Google..what? …* ” * (RD#1)*

*“Unfortunately these young ones that get in learn the hard way sometimes … It is not a job for the light hearted.” (RD#3)*

*“..it’s an interesting and unique clientele – I found it challenging and I loved it. In university there is a lot of discussion about disease states but not a lot of discussion about the different environments and how to deal with them and manage things.” (RD#6)*

“*The only time prisoners don’t eat in prison is due to segregation or if we have lock down … This year we’ve had three so far*. (reference is to number of lock downs up to February)” (*Stakeholder incarceration #16; SInc#16*)

Many expressed it was critical to have nutrition professionals that are both prepared and committed to working in corrections to prevent high turnover and burnout. Furthermore, there needed to be opportunities for professional growth:

*“It [the posting] has the duty for five years … they had a really hard time getting somebody long term … like for me personally … it’s kind of like I can’t do this job for that long if I don’t have some kind of outlet … ” (RD#1)*

*“ … you need to do a lot of self-directed stuff to work your way through it all … But really as far as keeping yourself up to date and really learning about the prison system we tend to look to the Americans … like the Correctional Foodservice and Nutrition Manual.” (RD#3)*

Contributing to the issues of burnout were the expectations to meet the diverse needs of incarcerated individuals in the many different facilities within a region. As voiced by one interviewee, you essentially “*hit the ground running”* and learn as you go.

Theme 2: Challenges with MNT Provision.

The main challenges with MNT provision included access, visibility, adequacy, and environmental barriers (Table 2). Within the coded narratives, interviewees highlighted examples of limited health service access which directly impacted MNT availability:

*“Access to health care is limited as it took a while before [incarcerated family member] was able to see the physician.” (Family Member #1; FM1)*

In conjunction with overall health service access issues, delays in access to MNT occurred due to factors such as clinic space constraints. Although on-site health clinics are available, appointments for nutrition counseling, even if requested by an incarcerated person, required a referral by a nurse or physician. Furthermore, if on a given day there was a surplus of health providers in the clinic, MNT would be cancelled in order to provide space.

**Table 2:** Summary of factors impacting delivery of corrections-based medical nutrition therapy in Canada (Theme 2)

1. Access	
System gate keeping	• Medical model system used for access to MNT (e.g., requirement for referrals) • Service delays due to clinic cancellations • Attempts to fill MNT voids by other health professionals
2. Visibility	
Lack of awareness about services	• Limited awareness of MNT by staff and incarcerated individuals
Misconceptions about services	• Tendency to view MNT as being about menu development
3. Adequacy	
Limited work time	• Low dietitian to incarcerated individuals ratio (e.g., one full-time equivalent dietitian to 2150 incarcerated individuals) • Services dispersed over many facilities with varying geographical proximities and diverse populations • Resource constraints (e.g., clinic space availability) creating delays in service
Skills development and utilization	• Post-secondary and entry-level nutrition training does not include correctional facilities • Limited therapeutic standards specific to incarcerated individuals (e.g., nutrition risk screening, assessment) • Underutilization of clinical nutrition skills
4. Environmental Barriers	
Food availability	• Options available to incarcerated individual vary by facility

Depending on the dietitian’s rotation among facilities within their region and competing demands, appointments for nutrition counseling could be significantly delayed:

“*… we had just one female prison and I only went there once in the whole time [7 years].” (RD#2).*

While group education sessions (e.g., food skills building) were considered to be important, these were conducted only if the topic was deemed necessary. Issues of nutrition service visibility and adequacy were also apparent. Some incarcerated individuals lacked awareness about MNT:

“*You’re telling me there is someone who looks after diet stuff? I never saw or heard anything about this..*” (*Incarcerated Individual*; from *Field Notes Document #10; FN#10*).

Some incarcerated individuals self-managed their nutrition care by selecting or preparing specific foods at mealtimes. One individual described how he supplemented his diet with various natural health products “*to feel vital and healthy*.” (*FN#16*).

Some incarcerated individuals were uncertain who to speak to about nutrition related issues. In some instances, incarcerated individuals knew they could access MNT, however, described them as inadequate. Similarly, some of the CSC staff indicated they did not fully understand the delivery of MNT within the facility:

*“There’s a doctor who comes. Sometimes physio. There is a nurse that works every day – does the meds. For other services like dietitian, orthotics, specialists they may need to go out.” (SInc8)*

In some regions, dietitians were responsible for providing their services to many different facilities and diverse populations (e.g., youth, adults, pregnant women, Aboriginals, older adults). Due to these high workloads, some worked “*more than full time even” (RD#1).* Similar sentiments were echoed about service adequacy:

*“It was hard to get anything really accomplished beyond 3 meals a day.” (RD#2)*

Beyond challenges with access, visibility, and adequacy were broader level contextual factors described by different stakeholders that impacted the implementation of MNT. For example, a family member discussed challenges about food access:

“*$35/week from CSC..allocated for food and beverages … and are not allowed to add more to it … can purchase [food] from the canteen from..own savings. [The Individual] had limited food skills..didn’t know a lot about how to prepare foods..wasn’t given any food skills training … .*” (*Family member: FM#1*)

Theme 3: Consideration of Corrections-Based MNT Alternatives.

Some stakeholders believed that MNT was being underutilized. Building food, nutrition, and health literacy skills were thought to be important for successful reintegration, however challenges in creating shifts in thinking about these service possibilities were noted:

*“It’s almost like they [inmates] see me as a service inside the institution only … when they get close to being released and getting out it’s like they don’t want to talk about diet anymore … They are more like ‘I am getting out of here, I will lose weight when I get out. I will control my blood sugar when I get out, everything will be fine when I get out’.” (RD#1)*

## Discussion

**Fig. 3:**
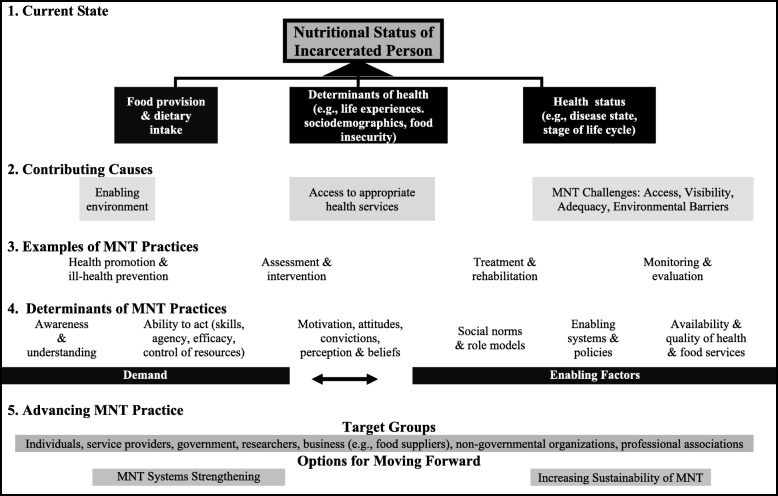
Working framework of pathways contributing to the nutritional status of incarcerated individuals with potential strategies for future medical nutrition therapy (MNT) practices

To place the thematic analysis in a broader context, a conceptual framework was developed to integrate the study’s findings with the pathways that lead to individual nutritional status (Fig. 3). Based on this framework and study findings, an incarcerated individual’s nutritional status depends upon an interplay of immediate factors that include dietary intake, the determinants of health, and health status (e.g., disease state, life stage) as well as underlying influences such as the environment and access to health services (Levels 1 and 2). In Level 2 of the model, MNT and its associated challenges of access, visibility, adequacy, and environmental barriers (Theme 2) are highlighted. Level 3 outlines examples of nutrition care practices; their success is subsequently impacted by: 1) demand for services which is dependent on awareness, motivation to act, and ability to act; and 2) enabling factors that include social norms and support, systems and policies, availability and quality of food, as well as health and support services (Level 4). The other parts of the framework shift to strategies that could help advance MNT delivery. Target groups and practice components that are identified in the model are described in the following.

### MNT systems strengthening

Clear system-wide policies and procedures for nutrition care staff, nutrition screening, referral, assessment, and education would help to improve continuity of care and prevent nutrition-related issues (e.g., malnutrition) and their associated complications in incarcerated individuals [75, 76]. As interviewees suggested, specific criteria about when they should be consulted and clear communication channels would enable consistent service:

*“ … I could leave notes for the physician, but they weren’t often in the institutions long … it was a different physician in different institutions … I would communicate through the health care chief.” (RD#2)*

Both the literature and stakeholder interview data highlighted how food choices are multi-factorial, even in correctional contexts [76].

*“..for example, in a grocery store where they place bags of chips how prominently they display it … how large of the percentage of the store is dedicated to certain types of foods … the cost of food … those choices as far as environment have impact both on habits … It’s the same in the prison system.. what’s available in the vending machines.. what’s available in the cafeteria..have long term impacts ..a second sort of factor is..motivation. We have certain motives for our food choices and I don’t think those motivations are in isolation … some people may make food choices based on their.. health goals – long range and short range – as well as what choices their friends and social peers are making and some of their ideology and beliefs … the environment that we create in the prison or outside the prison or the social circles and networks that are made are going to have major effects on the choices that are made*.” (*SInt#16*)

Different studies discuss that there is disconnect between what is specified on facility menus and the food that is consumed [11, 80]. This suggests that multiple stakeholders (e.g., facility and community-based, incarcerated individuals) need to be engaged to help implement appropriate strategies that target the multiple determinants of eating. For example, initiatives that are aimed at creating supportive food environments (e.g., improvements in food buy-up schemes, nutritional labeling on menus) and optimizing health and behavior (e.g., nutrition supplement studies in correctional facilities) have been implemented [11, 48, 80, 81].

Another fundamental gap identified by interviewees was the lack of specific evidence that would help to inform corrections-based nutrition practice. As an example, a dietitian asked about predisposing factors related to healthy weight management and responded:

“*That is an excellent question, and I don’t know. Some of them have always had weight issues, and they have continued to have weight issues, a lot of them didn’t and then gained a lot of weight in the institution, but this is just based on my observations. I don’t have info on that..*” (*RD#1*)

This need for evidence has also been identified in several studies worldwide [82]. In the literature there are very few specific examples (e.g., diabetes, pregnancy, tuberculosis, detoxification from chemical dependency, HIV, depression) [47, 68–72] of corrections-based MNT guidelines. Data such as the distribution of nutrition-related health conditions in the correctional systems, nutrition interventions that can improve behavior [80], and how to tailor services to address the needs of special populations (e.g., seniors) would help to better define types of nutrition services that are needed:

*“I also think that what is interesting is the needs of men and women are very different and the way they are managed. For women many have children they are getting back to and that is a goal.” (RD#8)*

### Increasing sustainability of MNT

Several viable alternatives are available that would help promote the sustainability of corrections-based MNT. For example, creating opportunities that would build knowledge about therapeutic diets among corrections staff and volunteers would better accommodate the needs of special populations (e.g., individuals with diabetes, pregnant women) [69, 70]:

*“There were like regular menus … other than that specific direction would have to be given for each individual [therapeutic] diet.” (RD#2)*

Interviewees also indicated that skills building for incarcerated individuals needed to be integrated more into their work:

*“ … It was about managing the present situation and side effects and stuff like that … I might help with budgeting and helping them select foods from the stores … we didn’t really talk a lot about skills..” (RD#6)*

*“ … We were getting to the point where they were wanting to learn about bread making, and whole wheat pita … ”*

In developed nations, there are many examples of food and nutrition skill building interventions in correctional facilities. Approaches such as training farms that build food literacy and skills of incarcerated individuals have been shown to substantially reduce food costs and improve employment potential of incarcerated individuals [52]. In some instances, the farms and gardens are located within the community and provide food for residents which aligns with the goals of restorative justice [90]. Examples from the Danish prison system illustrate how culinary education from chefs and self-sufficiency training enabled incarcerated individuals to shop and cook for themselves [82, 91].

Health promotion and wellness programs that are collaborative, use participatory and peer modeling approaches, and help build employment skills also help to optimize nutrition services as well as health and social outcomes [48, 82, 92]. For example, positive parenting programs that include healthy lifestyle interventions have led to returns on investment in excess of 6% through reduced use of various services including criminal justice [93].

*“ … that’s why we do peer support. Because we are people that have gotten out and we are trying to teach them about getting out. And things that we have found that are going to being successful on getting out.*.” *(SI#26 from focus group data)*

*“Any time we had an immigrant woman released from jail … They often taught the others [about cooking] and that worked out really well … ” (SI#31 from focus group data)*

Integrating different delivery alternatives would also enhance the sustainability of corrections-based MNT. Service delays could be reduced by using telehealth [94]. Interdisciplinary care conferencing, group education, and medical appointments could also create efficiencies [60, 95]. Collaborations between corrections and agencies that focus on societal reintegration would establish appropriate supports for ongoing nutrition care after incarceration. For example, a program that helped pregnant mothers who used substances prepare for nutrition care needs post-release improved their health outcomes [53].

*“Something should happen before release. It’s almost like a program you start three months before release that teaches life skills … How to grocery shop..How to cook..I think it’s hard when they are released they may have a place to go to but they don’t have money to get pots and pans … If you have acceptable housing I think there is potential to get some skills building after release … ” (RD#6)*

Finally, it must be recognized that future corrections-based MNT is dependent on preparing practitioners for this type of practice. Given that in some countries the vacancy rate of dietitians is approximately 40%, critical review of barriers impeding the pursuit of corrections-based employment is needed [18].

*“I think as dietitians these are not environments we are use to and I think it should be part of what we should learn.”(RD#6)*

As a starting point, utilizing successful models from other correctional settings to address dietitian skills training should be considered. For example, in Australia a service-learning model was adopted that partners government (prisons) and dietetic training programs that is sustainable and mutually beneficial [78]. Dietitians in-training work with nutrition professionals in federal prisons and in turn, the facilities receive students able to take part in hands-on delivery of clinical nutrition services. Over a 3 year trial period, the work of the students was equivalent to one full-time dietitian position [78]. Interprofessional training opportunities that enable learning with, from, and about others in corrections would help prepare nutrition professionals to be successful collaborators [57]. In addition, developing knowledge and skills in cultural competency [96, 97], trauma-informed care [98], harm reduction [99, 100], and equity-oriented care [101] are critical to working effectively in a corrections-based environment.

### Study limitations

While this has been the first study to provide in-depth exploration of MNT in Canadian correctional facilities, the investigative limitations must be noted. The process of bracketing (i.e., self-reflective process of recognizing and setting aside a priori knowledge and assumptions) employed in the original study was not applied in the secondary analysis of the data [102]. However, to minimize skewness in the reporting of results, all forms of data from the study were included and integrated with data from a broad scoping review. Selection bias was another limitation as the stakeholders were sampled based on their knowledge and experiences about food insecurity related to incarceration. However, conversations about food insecurity relied heavily on reflections about food and nutrition services in the corrections system and as part of the original sampling frame corrections-based food, nutrition, and health personnel were purposely selected. The individuals with experiences of incarceration who were interviewed may have been more health literate and may not have discussed issues that others face. The study was primarily conducted within one region of Canada and the results may not be generalizable to all provinces, territories, and incarcerated individuals. For example, few young individuals (e.g., < 18 years) and older adults were sampled. Further work in other regions, examining utilization data, and exploring experiences among more diverse groups would help to verify these findings.

## Conclusions

Having access to health care services such as MNT while incarcerated and during reintegration, along with other factors, can help lead to various societal benefits including reduced health care and social costs [103–109]. This secondary analysis of qualitative data helped shape an understanding about the delivery of corrections-based MNT in Canada. While there are many challenges associated with this type of work, there are also opportunities to better prepare incarcerated individuals for successful societal reintegration and reduce barriers that can contribute to marginalization and crime [109]. Professional associations, government, researchers and other stakeholders can all help to strengthen corrections-based MNT by creating shifts in thinking about the role of MNT in these contexts, preparing future practitioners with the specialized skills needed to work in these environments, generating evidence that can best inform practice, and fostering collaborations that can lead to successes in societal reintegration.

## Additional file

Additional file 1: Interview Guide, Interview Guides for Stakeholders and Persons with Lived Experience of Incarceration. (DOCX 17 kb)

## Abbreviations

FM: Family member
MNT: Medical nutrition therapy
RD: Registered Dietitian
SI: Stakeholder interview
